# Sequence of Polyurethane Ionomers Determinative for Core Structure of Surfactant–Copolymer Complexes

**DOI:** 10.3390/ijms22010337

**Published:** 2020-12-30

**Authors:** Elizabeth M. Timmers, Jose Rodrigo Magana, Sandra M. C. Schoenmakers, P. Michel Fransen, Henk M. Janssen, Ilja K. Voets

**Affiliations:** 1 Laboratory of Self-Organizing Soft Matter, Department of Chemical Engineering and Chemistry, Eindhoven University of Technology, 5600 MB Eindhoven, The Netherlands; e.m.timmers@tue.nl (E.M.T.); j.r.magana.rodriguez@tue.nl (J.R.M.); 2 Laboratory of Macro-Organic Chemistry, Department of Chemical Engineering and Chemistry, Eindhoven University of Technology, 5600 MB Eindhoven, The Netherlands; s.m.c.schoenmakers@tue.nl; 3 Institute for Complex Molecular Systems, Eindhoven University of Technology, 5600 MB Eindhoven, The Netherlands; 4 SyMO-Chem B.V., Den Dolech 2, 5612 AZ Eindhoven, The Netherlands; M.Fransen@TUE.NL (P.M.F.); H.Janssen@tue.nl (H.M.J.)

**Keywords:** micelle, block copolymer, co-assembly, precision polymers, polyurethane, scattering

## Abstract

The core of micelles self-assembled from amphiphiles is hydrophobic and contains little water, whereas complex coacervate core micelles co-assembled from oppositely charged hydrophilic polymers have a hydrophilic core with a high water content. Co-assembly of ionic surfactants with ionic-neutral copolymers yields surfactant–copolymer complexes known to be capable of solubilizing both hydrophilic and hydrophobic cargo within the mixed core composed of a coacervate phase with polyelectrolyte-decorated surfactant micelles. Here we formed such complexes from asymmetric (**PUI-A2**) and symmetric (**PUI-S2**), sequence-controlled polyurethane ionomers and poly(*N*-methyl-2-vinylpyridinium iodide)_29_-*b*-poly(ethylene oxide)_204_ copolymers. The complexes with **PUI-S2** were 1.3-fold larger in mass and 1.8-fold larger in radius of gyration than the **PUI-A2** complexes. Small-angle X-ray scattering revealed differences in the packing of the similarly sized PUI micelles within the core of the complexes. The **PUI-A2** micelles were arranged in a more ordered fashion and were spaced further apart from each other (10 nm vs. 6 nm) than the **PUI-S2** micelles. Hence, this work shows that the monomer sequence of amphiphiles can be varied to alter the internal structure of surfactant–copolymer complexes. Since the structure of the micellar core may affect both the cargo loading and release, our findings suggest that these properties may be tuned through control of the monomer sequence of the micellar constituents.

## 1. Introduction

Micelles are ubiquitous in products from the food, pharmaceutical, and coatings industries, and the assembly properties of micelles have been studied extensively [1,2,3]. Most core–shell micelles are self-assembled in aqueous solution from small molecular or polymeric amphiphiles driven by the hydrophobic effect. Their properties are mainly governed by the length of the hydrophobic block, the presence or absence of charges in the hydrophilic block, and the chain architecture [4,5]. Classical core–shell micelles, composed of small molecular or polymeric amphiphiles, typically have a densely packed hydrophobic core with a low water content and can be used to encapsulate hydrophobic cargo [2,3]. Polymeric micelles often display a frozen structure; very little exchange of chains between micelles and the medium takes place [6]. Polymeric micelles with a comparatively large core are referred to as crew-cut micelles, whereas micelles with a large corona and small core are described as star-like [7]. Contrary to these micelles composed of amphiphiles, complex coacervate core micelles (C3Ms) can readily solubilize hydrophilic cargo [8]. C3Ms are assembled from fully water-soluble polymers [8], for example, from a double hydrophilic diblock copolymer composed of a neutral and a charged block and an oppositely charged homopolymer. The core consists of a coacervate phase with a high water content and is stabilized against macroscopic phase separation by the neutral corona [8]. C3Ms composed of weak polyelectrolytes with a pH-dependent charge density are responsive to variations in both pH and ionic strength. Interestingly, the salt concentration can be tuned to prevent kinetically trapped architectures and promote dynamical exchange of polymers between micelles [9].

Micelles capable of solubilizing both hydrophilic and hydrophobic cargo can be readily prepared upon co-assembly of amphiphiles with oppositely charged double hydrophilic block copolymers to give surfactant–copolymer complexes [10]. This is because the core of the finite-sized colloids, which form if the neutral block of the copolymer is sufficiently long, now contains hydrophobic segments embedded within a coacervate phase. The formation of surfactant–copolymer complexes is driven by both hydrophobic and electrostatic interactions, offering a multitude of handles to tune the assembly properties. These include, for example, the charge ratio between oppositely charged components [11,12] and the degree of polymerization and block length ratio [13], as well as the temperature, ionic strength, and pH [14]. Solomatin et al. assessed the response to changes in ionic strength and pH of surfactant–copolymer complexes and deemed such complexes suitable for drug delivery applications [15,16]. Furthermore, they found stronger ordering of the surfactants within the core of the complexes compared to surfactant micelles alone [17]. Voets et al. established surfactant-copolymer complexes when elevated temperatures caused poly(acrylic acid)-block-poly(isopropyl acrylamide) to become amphiphilic and collapse whilst stabilized by double hydrophilic diblock copolymers [14]. Balomenou and coworkers applied grafting of neutral hydrophilic chains to a polyelectrolyte backbone to prevent macroscopic phase separation and achieve finite-sized complexes upon co-assembly with dodecyl trimethylammonium bromide [18]. Chen et al. showed that the overall size of the complexes was determined by the poly(ethylene oxide) (PEO) grafting density, where higher PEO content led to smaller sizes [19]. Vitorazi et al. found that (a) symmetry in the block length ratio of the double hydrophilic diblock copolymer strongly affected the core. Asymmetric ratios, with a longer neutral block than the charged block, led to a disordered surfactant micelle core, whereas symmetry in the block lengths yielded liquid crystalline ordering in the core [20]. Berret et al. found that the charge ratio determined whether the complexes are single surfactant micelles decorated with a few copolymers or objects with a core of many densely packed surfactant micelles and a shell of the neutral block [11].

The possibility of packaging both hydrophilic and hydrophobic cargo and the modularity of the core structure render surfactant–copolymer complexes interesting candidates for the encapsulation of various compounds, including drugs. The impact of core structure on drug delivery and release has not yet been addressed for surfactant–copolymer complexes, but it has been intensely investigated for other types of polymeric micelles aiming to increase drug loading and slow down drug release. Drug loading capacity can be increased by tuning the stereostructure [21] or by stereocomplexation [22] in the core, by optimizing the solubility parameter of the hydrophobic block towards the drug [23,24], by reducing core crystallinity [25], or by decreasing the core density (e.g., via a rigid core block) [26]. Similarly, sustained release can be achieved by tuning the stereostructure of the core-forming block [21], stereocomplexation in the core [27], increasing core crystallinity [28], tuning the core density through variations in the architecture of the hydrophobic block [22], or increasing the acyl chain length [24]. Additionally, the architecture of the neutral PEO block can be used to improve both loading and release [29]. Thermo-responsive constituents can be incorporated to trigger drug release [23]. Yang et al. found slower drug release for a triblock compared to diblock structure, which was attributed to a higher density of the triblock micellar core [22].

**Figure 1:**
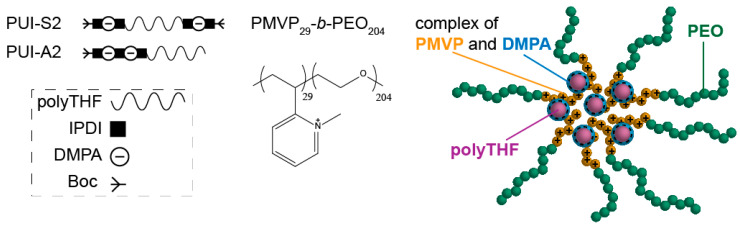
Proposed structure of a surfactant–copolymer complex co-assembled from a sequence-controlled symmetric polyurethane ionomer (**PUI-S2**) or asymmetric PUI (**PUI-A2**) (chemical structures in Figure A1) with double hydrophilic diblock copolymer poly(N-methyl-2-vinylpyridinium iodide)_29_-*b*-poly(ethylene oxide)_204_ (PMVP_29_-*b*-PEO_204_). PUIs are composed of poly(tetrahydrofuran) (polyTHF), isophorone diisocyanate (IPDI), dimethylpropionic acid (DMPA), and tert-butyloxycarbonyl (Boc). The core–shell surfactant–copolymer complexes contain a core comprising the anionic PUI micelles associated with the cationic PMVP segments of the block copolymer.

Inspired by these studies on the relation between constituent architecture and core structure, we herein investigate the impact of the monomer sequence of the amphiphile on the core structure of surfactant–polymer complexes. Recently, we reported micellization of two sequence-controlled polyurethane ionomers (PUIs) with an asymmetric anionic–hydrophobic structure (resembling a classical surfactant or diblock copolymer) and its symmetric counterpart (resembling a bolaamphiphile or triblock copolymer) [30]. Here, we studied the co-assembly of either of the anionic PUIs with the cationic-neutral poly(*N*-methyl-2-vinylpyridinium iodide)_29_-*b*-poly(ethylene oxide)_204_ (PMVP_29_-*b*-PEO_204_) (Figure 1). We characterized the size, shape, and structure of these surfactant–copolymer complexes using light and X-ray scattering and cryogenic transmission electron microscopy. We thereby mapped the properties of these complexes with a virtually identical composition but differing only in PUI sequence to assess sequence effects on co-assembly.

## 2. Results

Aiming to study the impact of surfactant sequence on the properties of surfactant–copolymer complexes, association colloids were first prepared upon mixing the double hydrophilic block copolymer with the sequence-controlled polyurethane ionomers (PUIs). To this end, an aqueous solution of PMVP_29_-*b*-PEO_204_ in 10 mM NaNO_3_ was mixed with a solution in tetrahydrofuran (THF) of either the asymmetric **PUI-A2** or the symmetric **PUI-S2** to yield an aqueous–organic micellar solution with a 0.1 THF volume fraction and total polymer concentration of 10 mg/mL.

**Figure 2:**
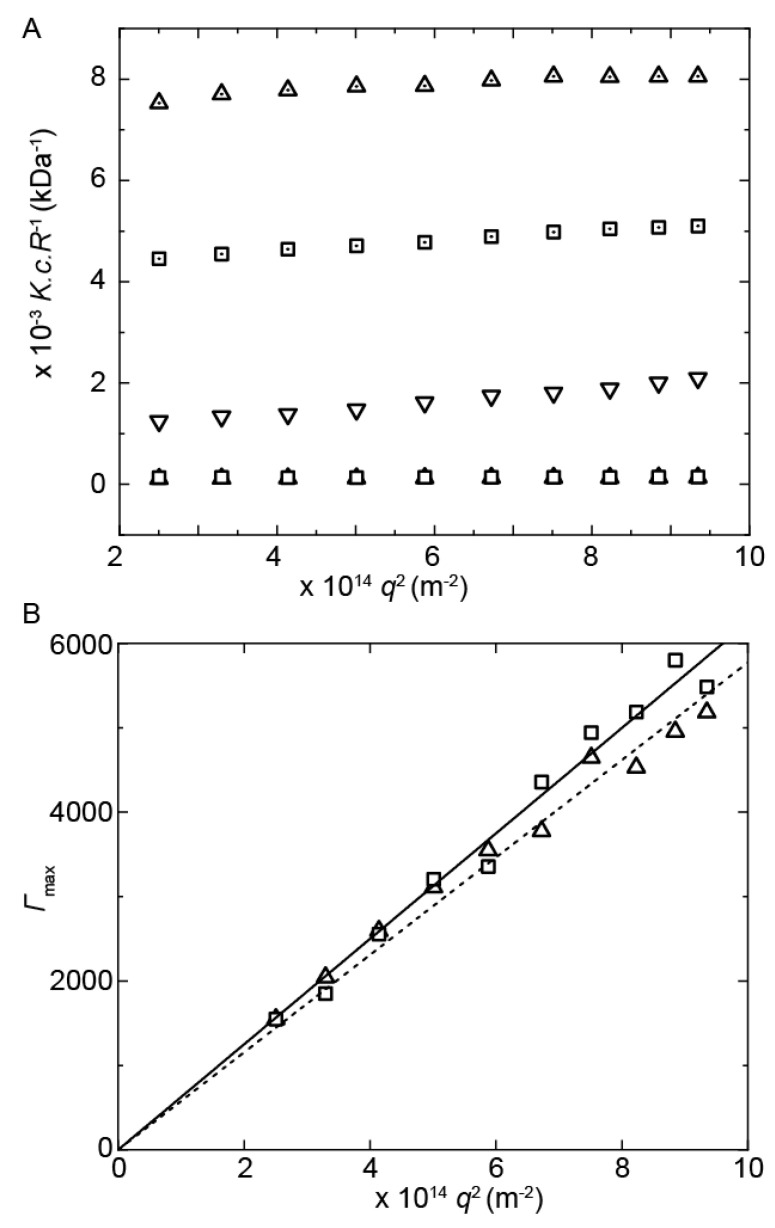
(**A**) Partial Zimm plot of separate constituents and PUI:PMVP_29_-*b*-PEO_204_ complexes constructed to compare the Rayleigh ratio, *R*(q), and to obtain *M*_complex_ and *R*_g_ for PUI:PMVP_29_-*b*-PEO_204_ from the linear fits (not shown). (**B**) Mean frequency *Γ* from multi-angle dynamic light scattering (DLS) vs. *q*^2^ for PUI:PMVP_29_-*b*-PEO_204_ complexes; lines represent linear fits. **PUI-A2** at *φ*_THF_ = 0.1 in water (dotted square), **PUI-S2** at *φ*_THF_ = 0.1 in water (dotted triangle), PMVP_29_-*b*-PEO_204_ at *φ*_THF_ = 0.1 in 10 mM NaNO_3_ (downwards pointed triangle), and mixtures of **PUI-A2**:PMVP_29_-*b*-PEO_204_ (square) and **PUI-S2**:PMVP_29_-*b*-PEO_204_ (triangle) at *φ*_THF_ = 0.1 in 10 mM NaNO_3_ with a total polymer concentration of 10 mg/mL.

Static light-scattering experiments revealed a 107-fold and 229-fold increase in the Rayleigh ratio, *R*(q), for **PUI-A2** and **PUI-S2**, respectively, upon mixing with the diblock, which signals the formation of complexes with a mass much larger mass than that of the individual constituents (Figure 2A). The apparent mass and aggregation number of the surfactant–copolymer complexes were determined from the angular dependence of *R*(q) at a fixed polymer concentration across a scattering wave vector range of 0.015 < *q* < 0.031 nm^−1^. Notice that these measurements were performed at a single concentration to avoid concentration-induced variations in aggregation number and therefore we cannot rule out contributions of interparticle interactions. The contrast term (dn/dc) is an estimated value based on literature dn/dc values of comparable compounds. The computed apparent molecular weights may therefore deviate from the actual molecular weight. Guinier calculations gave radii of gyration, *R*_g_, of 19 nm for **PUI-A2**:PMVP_29_-*b*-PEO_204_ and 34 nm for **PUI-S2**:PMVP_29_-*b*-PEO_204_. Similarly, the complexes with **PUI-S2** had a 1.3 times larger apparent molecular weight (9804 kDa) compared to **PUI-A2**:PMVP_29_-*b*-PEO_204_ (7800 kDa). The aggregation numbers, *N*_agg_, of the individual constituents in the complex were determined under the assumption that the overall complex is charge-neutral and composed of many copies of subunits comprising the smallest possible charge-neutral unit with a mass *M*_unit_. This computation revealed that the complexes contained several thousand polyurethanes and several hundred diblock copolymers in a weight ratio of approximately 3:1. Specifically, we found lower aggregation numbers for PUI-A2 complexes (ca. *N*_agg, PUI_ = 1790 and *N*_agg, dbp_ = 190) compared to PUI-S2 (ca. *N*_agg, PUI_ = 2109 and *N*_agg, dbp_ = 224).

**Figure A2:**
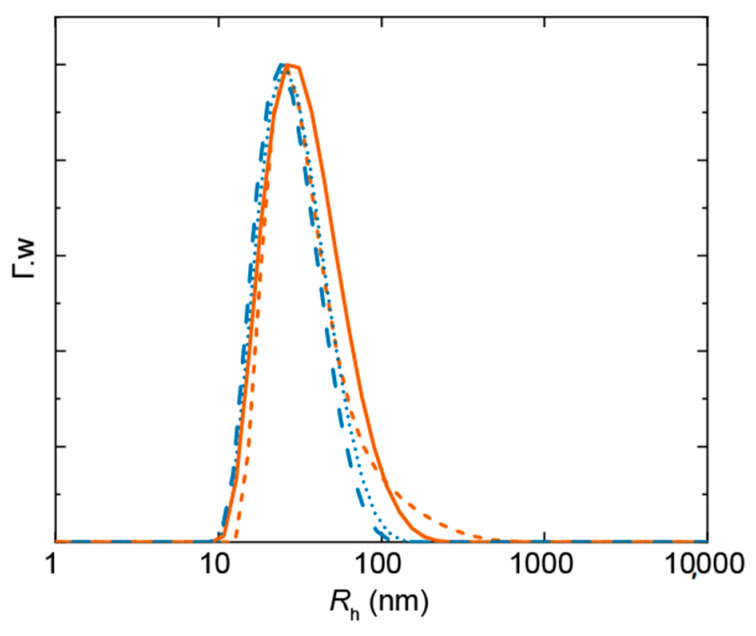
Size distributions from Contin analysis at 90° from DLS of **PUI-S2**:PMVP_29_-PEO_204_ (orange) and **PUI-A2**:PMVP_29_-PEO_204_ (blue) freshly prepared (dotted and dash) and after 7 days of storage (solid and long dash).

The static light-scattering experiments were complemented with multi-angle dynamic light-scattering experiments (0.015 < *q* < 0.031 nm^−1^) to measure the apparent hydrodynamic radii, *R*_h_, and *R*_g_/*R*_h_ ratios of the complexes. Monomodal frequency (*Γ*) distributions were obtained from a CONTIN analysis of the second-order correlation functions, *g*_2_ (t). No significant changes in these size distributions were observed up to at least 7 days after preparation (Figure A2). The mean apparent diffusion coefficients, *D*_app_, were subsequently determined from the slope of the linear dependence of *Γ* on *q*^2^ (Figure 2B), and from the corresponding *R*_h_ using the Stokes–Einstein relation. The hydrodynamic radius of the **PUI-S2** complexes was slightly larger with *R*_h_ = 28 nm and *R*_h_ = 26 nm for **PUI-S2**:PMVP_29_-*b*-PEO_204_ and **PUI-A2**:PMVP_29_-*b*-PEO_204_, respectively. These micellar sizes were similar to those previously reported for spherical complex coacervate core micelles (C3Ms) with PMVP-*b*-PEO copolymers and oppositely charged species [31]. For example, an *R*_h_ of approximately 20 nm was found for spherical C3Ms comprising poly (acrylic acid) [32] and tetrakis (4-carboxyphenyl) porphyrin [33] bound to PMVP_41_-*b*-PEO_204_ diblock copolymers with a slightly larger core:corona block length ratio than the PMVP_29_-*b*-PEO_204_ copolymers used herein. C3Ms comprising the two diblocks PMVP_38_-*b*-PEO_211_ and poly (acrylic acid)-*b*-poly(isopropyl acrylamide) were slightly smaller still with a *R*_h_ = 14 nm at room temperature [14].

**Figure 3:**
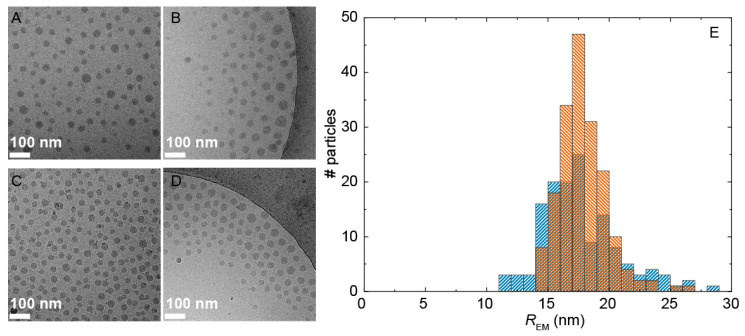
(**A**–**D**) Representative cryoTEM micrographs of **PUI-A2**:PMVP_29_-*b*-PEO_204_ with (**A**) and without (**B**) uranyl acetate staining, and of **PUI-S2**:PMVP_29_-*b*-PEO_204_ without staining (**C**,**D**). (**E**) Size distributions showing the number (#) of particles obtained from image A–D, *n* = 140 for **PUI-A2**:PMVP_29_-*b*-PEO_204_ (blue ///) and *n* = 180 for **PUI-S2**:PMVP_29_-*b*-PEO_204_ (orange \\\).

Cryogenic transmission electron microscopy (cryoTEM) experiments were performed next to visualize the complexes to examine their morphology and determine the mean dimensions of the micellar core (the corona was generally invisible due to its low contrast). We attempted to increase the core contrast by staining with uranyl acetate. However, we found no significant difference between stained and unstained samples (see for example Figure 3A vs. Figure 3B). As expected, spherical structures were observed in the electron micrographs of both systems (Figure 3). Interestingly, for both **PUI-A2**:PMVP_29_-*b*-PEO_204_ (Figure 3A,B) and **PUI-S2**:PMVP_29_-*b*-PEO_204_ (Figure 3C,D) we measured an average radius, *R*_EM_, of 18 nm (Figure 3E). This gave a shell thickness, defined as the difference between *R*_h_ and *R*_EM_, of 10 and 8 nm for complexes with **PUI-S2** and **PUI-A2**, respectively. The core of both mixed micellar complexes was thus considerably larger than the corona. CryoTEM further revealed a high similarity in the size distributions of the micellar cores (Figure 3E), from which we computed a modest core dispersity *Ð*_EM_ = 0.11 (**PUI-S2**) and 0.18 (**PUI-A2**).

**Figure 4:**
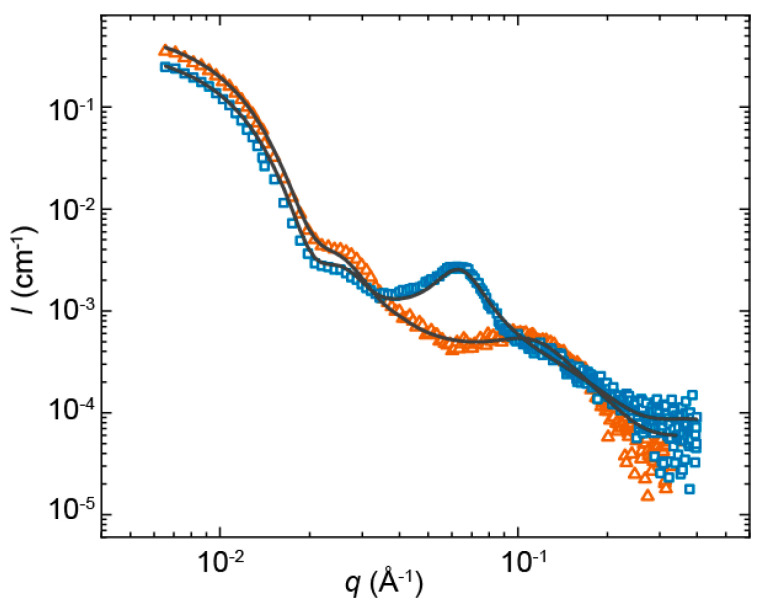
Measured small-angle X-ray scattering (SAXS) profiles of **PUI-S2**:PMVP_29_-*b*-PEO_204_ (open triangle, orange) and **PUI-A2**:PMVP_29_-*b*-PEO_204_ (solid square, blue), and computed models (solid line).

Having established the morphology and dimensions of the surfactant–copolymer complexes, small-angle X-ray scattering (SAXS) experiments were performed for 0.006 Å^−1^ < *q* < 0.4 Å^−1^ to study the internal morphology of the mixed micelles. Both SAXS profiles displayed a well-defined interference pattern reminiscent of experimental [11,34] and computed [35] SAS patterns previously reported by others for surfactant–copolymer complexes (Figure 4). Two main structural features stood out. In the low-*q* regime for *q* < 0.03 Å^−1^, we observed strong forward scattering related to the spherical overall shape of the complexes. In the high-*q* regime for *q* > 0.03 Å^−1^, the profiles were dominated by the scattering contribution of the PUI micelles within the mixed micellar core and the correlation peak associated with the inter-PUI-micellar distance. The latter contribution was more pronounced for complexes with **PUI-A2** than **PUI-S2**.

Previous work has ascribed such a peak to dense packing of surfactant micelles within the core of surfactant–copolymer complexes, due to their confinement and increased local concentration [11,13,35,36]. In this work, we used a simplified model, reported in [37], to fit the experimental curves comprising a spherical form factor to account for the morphology of the overall complex (sphere 1), and a spherical form factor to describe the shape of the PUI micelles (sphere 2). To account for the structure factor arising from inter-PUI-micellar interactions we included a Lorentzian peak function. The sum model then became:

$$\begin{array}{c} I\left(q\right)=q*V_{1}*\Delta \rho_{1}^{2}*sc_{1}*{\left[\frac{\sin\left(q*r_{1}\right)-q*r_{1}*\cos\left(q*r_{1}\right)}{q^{3}*r_{1}^{3}}\right]}^{2}\;\;\;\;\;\;\;\;\;\;\;\;\;\;\;\;\;\;\;\\ +q*V_{2}*\Delta \rho_{2}^{2}*sc_{2}*{\left[\frac{\sin\left(q*r_{2}\right)-q*r_{2}*\cos\left(q*r_{2}\right)}{q^{3}*r_{2}^{3}}\right]}^{2}+I_{\max}*\frac{\sigma^{2}}{4*\left(q-q_{\max}\right)+\sigma^{2}}+bkg \end{array}$$

where *V* is the volume of the scatterer, Δ*ρ* is the difference in scattering length densities (SLD) of the solvent and the scatterer, *sc* is the aggregate volume fraction, *r*_1_ is the radius of the overall complex, *r*_2_ is the radius of the PUI micelles, and *bkg* is the background scattering. *I*_max_, *q*_max_, and *σ* correspond to the maximum, the center, and the full-width at the half-maximum (FWHM) of the Lorentzian expression, respectively. *q*_max_ and σ are related to the distance between PUI micelles [35], *d*, and correlation length of the periodic arrangement of the micelles, describing over how many length scales the order decays, *ξ*, by the following equations:

$$d=\frac{2*\pi }{q_{\max}}$$

And

$$\xi =\frac{1}{\sigma }$$

The correspondence between the experimental SAXS data and the model was excellent (Figure 4). We found an overall radius of approximately 20 nm for the PUI:PMVP_29_-*b*-PEO_204_, in agreement with the light scattering and electron microscopy results. The dispersity of the overall complexes (ca. 0.2) was introduced into the model by averaging the form factor of sphere 1 over a Schultz distribution. The radii of the PUI micelles within the core of the complexes were roughly 1.4 nm, which is in good agreement with *R*_h_ ≈ 3.8 nm previously reported for the PUI micelles in solution in absence of diblock copolymer at *φ*_THF_ = 0.1 [30]. The **PUI-S2** micelles were packed more tightly in the core of the complexes, as evidenced by the smaller apparent intermicellar distance *d* = 6 nm for **PUI-S2** micelles as compared to *d* = 10 nm for **PUI-A2**. We propose that this is because there might be a larger number of PUI-S2 micelles within the core as bolaamphiphilic architectures are known to have lower aggregation numbers and therefore their chains are distributed amongst more micelles. This is in line with the lower aggregation numbers previously reported for **PUI-S2** compared to **PUI-A2** micelles in solution [30]. Interestingly (and presumably the biggest difference between both PUI:PMVP_29_-*b*-PEO_204_ complexes), the periodical arrangement of the polyurethane micelles within the complex core was much more pronounced for the asymmetric **PUI-A2**, which gives rise to a 2.7-fold larger correlation length *ξ* of 6.7 nm (**PUI-A2**) vs. 2.5 nm (**PUI-S2**). This distinctly different ordering might have important implications for drug delivery applications, as the packing density and ordering within micellar cores affects both drug loading [21,22,25,26] and release [21,22,27,28].

## 3. Materials and Methods

### 3.1. Materials

The synthesis and characterization of the two sequence-controlled polyurethane ionomers (PUI) through a stepwise coupling-deprotection approach to obtain the symmetric PUI (**PUI-S2**) and the asymmetric PUI (**PUI-A2**) is described in previous work [30]. The PMVP_29_-*b*-PEO_204_ was kindly supplied by Dr. Hande Cingil, quaternized from commercially available poly(2-vinylpyridinium iodide)-b-poly(ethylene oxide) as described in previous work with 65% functionalization [38].

### 3.2. Sample Preparation

The polymer–surfactant complexes were prepared by mixing 0.1 mL of a 71.9 mg/mL **PUI-S2** or 70.1 mg/mL **PUI-A2** solution in tetrahydrofuran (THF) containing triethylamine (TEA) in a 2:1 molar ratio of TEA:DMPA groups with 0.9 mL of a filtered sample containing 3.11 or 3.32 mg/mL PMVP_29_-*b*-PEO_204_ (for the two respective PUI types) in aqueous 10 mM NaNO_3_. The final samples contained a total PUI + diblock copolymer concentration of 10 mg/mL with a 9.425:1 molar ratio of PUI:PMVP_29_-*b*-PEO_204_ (about 3:1 in weight) and a THF volume fraction of 0.1. The final solution had a number of cationically chargeable monomers with respect to the total chargeable monomers of 0.56, and the overall charge ratio at the alkaline pH used in this work was PUI:diblock 1:0.83 for **PUI-S2** and 1:0.84 for **PUI-A2** complexes.

### 3.3. Static Light Scattering (SLS) and Dynamic Light Scattering (DLS)

SLS and DLS measurements were performed on an ALV Compact Goniometer System (CGS-3) instrument equipped with an ALV-7004 Digital Multiple Tau Real Time Correlator and a 40-mW solid state laser operating at a wavelength *λ* = 532 nm. The second-order correlation function, *g*_2_(t), and total mean scattered intensity, *I*, were recorded at scattering vectors (*q*) of 0.015 < *q* < 0.031 nm^−1^ in three runs of 30 s each, with *q* defined as:

(1) $$q=4*\pi *n_{s}*\frac{\sin\frac{\theta }{2}}{\lambda }$$

with the scattering angle, *θ*, and solvent refractive index *n*_s_ = 1.34 [30]. After ALV software (Dullware Inc.) based on the CONTIN algorithm was used to determine the frequency *Γ* from the mean g_2_(t), the apparent translational diffusion coefficient, *D*^app^, was obtained from a linear fit of *Γ* as:

(2) $$\Gamma =D^{\mathrm{app}}*q^{2}.$$

Using the Stokes–Einstein equation, we determined the apparent hydrodynamic radius, *R_h_*, from *D*^app^:

(3) $$R_{h}^{}=\frac{k_{B}*T}{6*\pi *\eta *D^{\mathrm{app}}}$$

Where *k*_B_ and *T* are the Boltzmann constant and temperature, and the viscosity *η* = 1.329 mPa*s [30]. The Rayleigh ratio, *R*, was calculated from the measured scattered intensity by:

(4) $$R=\frac{(I_{s}-I_{\mathrm{bkg}})*n_{s}^{2}}{\left(I_{t}-I_{\mathrm{dc}}\right)*n_{t}^{2}}*R_{t}$$

where *I*_s_, *I*_t_, *I*_bkg_, and *I*_dc_ are the scattering intensities of the sample, toluene reference, solvent, and dark current, respectively, *n*_t_ is the refractive indices of toluene, and *R*_t_ is the Rayleigh ratio of toluene (*R*_t_ = 2.07 × 10^−5^ cm^−1^) [39]. *I*_bkg_ and *I*_dc_ were assumed negligibly small in comparison to *I*_s_ and *I*_t_.

The apparent mass of the surfactant-copolymer complexes (*M*_complex_) and radius of gyration (*R*_g_) were obtained from the Zimm equation:

(5) $$\frac{K*c}{R}=\frac{1}{M_{\mathrm{complex}}}*\left(1+\frac{q^{2}*R_{g}^{2}}{3}\right)$$

with polymer concentration (*c*) and the contrast term (*K*), which was computed from:

(6) $$K=\frac{4*\pi^{2}*n^{2}*{\left(\frac{dn}{dc}\right)}^{2}}{N_{A}*\lambda^{4}}$$

with Avogadro’s number (*N*_A_) and the refractive index increment (*dn*/*dc*), which was approximated from the *dn*/*dc* of each component (*dn*/*dc_PUI_* = 0.135 cm^3^/g estimated from Rasolonjatovo et al. based on the pTHF chain [40], *dn*/*dc_PEO_* = 0.124 cm^3^/g [41], *dn*/*dc_PMVP_* = 0.204 cm^3^/g [42]) as:

(7) $$\frac{dn}{dc}=w_{PUI}*{\frac{dn}{dc}}_{PUI}+w_{PEO}*{\frac{dn}{dc}}_{PEO}+\;w_{PMVP}*{\frac{dn}{dc}}_{PMVP}$$

with *w* being the weight fraction of each component; additive *dn*/*dc* = 0.138 cm^3^/g for both systems. The apparent aggregation number (*N*_agg_) was obtained from the *N*_agg_ = *M*_complex_/*M*_unit_ where *M*_unit_ represents the weight of one charge-neutral complex (theoretically composed of 1 PMVP_29_-*b*-PEO_204_ chain and 9.425 PUI chains).

### 3.4. Small-Angle X-ray Scattering (SAXS)

SAXS experiments were performed on a SAXSLAB Ganesha instrument equipped with a GeniX-Cu ultra-low divergence source that produces X-ray photons with *λ* = 1.54 Å and with a flux of 1*10^8^ photons/s. A Pilatus 300K silicon pixel detector was used to collect scattering patterns in the range 0.006 Å^−1^ < q < 0.4 Å^−1^. Each sample was measured in 2-mm quartz capillaries for 7 h in two configurations. The resulting 2D images were radially averaged using SAXSgui software to obtain the intensity (*I*_SAXS_) vs. q profiles. Additionally, SAXSutilities was employed for data reduction procedures such as subtraction of the scattering of the solvent. The processed curves were modeled using SasView software, combining the sphere form factor with a Lorentzian function (details in Appendix A).

### 3.5. Cryogenic Transmission Electron Microscopy (cryoTEM)

Quantifoil grids (R 2/2, Quantifoil Micro Tools GmbH) were surface plasma-treated with a Cressington 208 carbon coater for 40 s at 5 mA just prior to use. Samples for CryoTEM imaging at 10 mg/mL total polymer concentration, THF volume fraction of 0.1, with and without 0.02% uranyl acetate were vitrified using a computer-controlled vitrification robot (FEI Vitrobot^TM^ Mark IV, FEI Company, Hillsboro, OR, USA). During vitrification the Vitrobot operated at 22 °C and at a relative humidity of 100%. In the preparation chamber of the ‘Vitrobot’, a 3-μL sample was applied on the Quantifoil grid. Excess sample was removed by blotting using two filter papers for 3 s with a blotting force of –1, and the thin film thus formed was plunged (acceleration about 3 g) into liquid ethane just above its freezing point. Vitrified films were transferred into the vacuum of a CryoTITAN equipped with a field emission gun that was operated at 300 kV, a post-column Gatan energy filter, and a 2048 × 2048 Gatan CCD camera and observed at temperatures below −170 °C. Micrographs were taken in low-dose conditions, starting at a magnification of 6500 with a defocus setting of 40 µm, and at a magnification of 24,000 with a defocus setting of either 5 or 10 µm. The radius (*R_EM_*) of the objects was measured using ImageJ software and the dispersity obtained through:

(8) $${Ð}_{EM}=\frac{SD}{mean}$$

from the standard deviation, *SD*, and mean size. Additionally, the shell thickness was estimated from:

(9) $$R_{shell}=\;R_{h}-R_{EM}$$

## 4. Conclusions

In this work, we co-assembled asymmetric (**PUI-A2**) or symmetric (**PUI-S2**) sequence-controlled PUIs and PMVP_29_-*b*-PEO_204_ diblock copolymers into surfactant–copolymer complexes to study the effect of the PUI sequence on the assemblies. We applied DLS, SLS, SAXS, and cryoTEM to characterize the complexes with a neutral PEO corona and a core composed of a coacervate phase with PUI micelles. The surfactant–copolymer complexes with **PUI-S2** were 1.3-fold larger in mass and their *R*_g_ was 1.8-fold larger than for complexes with **PUI-A2**. Additionally, modeling of the SAXS profiles revealed distinctly different ordering within the core of the complexes. The **PUI-A2** micelles were spaced wider apart as the PUI chains were distributed amongst fewer micelles. Interestingly, the **PUI-A2** were packed in a more orderly fashion, giving rise to a more pronounced structure peak and longer correlation length *ξ*. We attribute these effects to the marked difference in monomer sequence in **PUI-A2** and **PUI-S2**. Since the core structure of drug delivery systems impacts cargo loading and release, our findings offer an interesting avenue for further research exploring whether encapsulation and release from surfactant-polymer complexes may be fine-tuned through control of the monomer sequence in the surfactant component.

## Figures and Tables

**Figure A1:**
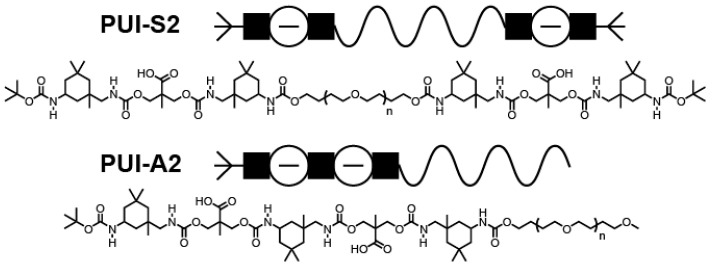
Chemical structures of **PUI-S2** and **PUI-A2**. The molecular weight (M_n_) of the polyTHF block in both PUIs is about 2 kDa.

## Data Availability

Data is contained within the article or Appendix A.

## Abbreviations

PUI	Polyurethane ionomer
PMVP_29_-*b*-PEO_204_	Poly(*N*-methyl-2-vinylpyridinium iodide)_29_-*b*-poly(ethylene oxide)_204_
THF	Tetrahydrofuran
DLS	Dynamic light scattering
SLS	Static light scattering
SAXS	Small-angle X-ray scattering
cryoTEM	Cryogenic transmission electron microscopy

## Appendix A

**SAXS modeling**. Curves were modeled using SasView software, by summing a spherical form factor [43] to represent the surfactant–copolymer complex (r_1_) with a second spherical form factor [43] to represent the PUI micelles in the core (r_2_) combined with a Lorentzian function to represent the intermicellar distance. The full set of modeling parameters is reported in Table A1.

**Table A1:** SAXS modeling parameters: scale (*sc*), background scattering (*bkg*), scattering length density (SLD), dispersity (*Ð*), and the maximum (*I*_max_), center (*q*_max_), and half-width at half-maximum (*σ*) of the Lorentzian function.

System	PUI-S2:PMVP_29_-*b*-PEO_204_	PUI-A2:PMVP_29_-*b*-PEO_204_
*sc*_1_	1 × 10^−3^	1 × 10^−3^
*bkg* (cm^−1^)	1 × 10^−4^	1 × 10^−4^
SLD r_1_ (Å^−2^)	330.4	330.3
SLD_solvent_ r_1_ (Å^−2^)	330	330
r_1_ (nm)	20	20
*Ð*_SAXS_ r_1_	0.17	0.17
*sc*_2_	4 × 10^−3^	4 × 10^−3^
SLD r_2_ (Å^−2^)	330.4	330.3
SLD_solvent_ r_2_ (Å^−2^)	330.6	330.6
r_2_ (nm)	1.5	1.3
*I*_max_	3.0 × 10^−4^	21 × 10^−4^
*q*_max_ (Å^−1^)	0.108	0.061
*σ*	0.04	0.015
